# Treatment of Common Cold Patients with the Shi-Cha Capsule: A Multicenter, Double-Blind, Randomized, Placebo-Controlled, Dose-Escalation Trial

**DOI:** 10.1155/2012/254571

**Published:** 2012-12-27

**Authors:** Jing Chang, Shou-Jin Dong, Bin She, Rui-Ming Zhang, Mao-Bin Meng, Yan-Ling Xu, Li-Ling Wan, Ke-Hua Shi, Jun-Hun Pan, Bing Mao

**Affiliations:** ^1^Pneumology Group, Department of Integrated Traditional Chinese and Western Medicine, West China Hospital, West China School of Medicine, Sichuan University, 37 Guoxue Lane, Sichuan Province, Chengdu 610041, China; ^2^Division of Clinical Pharmacology, West China Hospital, West China School of Medicine, Sichuan University, 37 Guoxue Lane, Sichuan Province, Chengdu 610041, China; ^3^Department of Radiation Oncology, Tianjin Medical University Cancer Institute & Hospital, Tianjin 300060, China; ^4^Department of Respiratory Medicine, Affiliated Hospital of Liaoning University of Traditional Chinese Medicine, Shenyang 110032, China; ^5^Department of Respiratory Medicine, Affiliated Hospital of Jiangxi College of Traditional Chinese Medicine, Nanchang 330006, China; ^6^Department of Respiratory Medicine, Shanghai Traditional Chinese Medicine Hospital, Shanghai 200071, China; ^7^Department of Respiratory Medicine, The First Affiliation Hospital of Guangzhou Medical University, Guangzhou 510405, China

## Abstract

This study was designed to determine the therapeutic efficacy and safety of the Shi-cha capsule, a Chinese herbal formula, in the treatment of patients with wind-cold type common cold. In our multi-center, prospective, double-blind, randomized, placebo-controlled, dose-escalation trial, patients with wind-cold type common cold received 0.6 g of Shi-cha capsule plus 0.6 g placebo (group A), 1.2 g of Shi-cha capsule (group B), or 1.2 g placebo (group C), three times daily for 3 days and followed up to 10 days. The primary end point was all symptom duration. The secondary end points were main symptom duration, minor symptom duration, the changes in cumulative symptom score, main symptom score, and minor symptom score 4 days after the treatment, as well as adverse events. A total of 377 patients were recruited and 360 met the inclusive criteria; 120 patients constituted each treatment group. Compared with patients in group C, patients in groups A and B had significant improvement in the all symptom duration, main symptom duration, minor symptom duration, as well as change from baseline of cumulative symptom score, main symptom score, and minor symptom score at day 4. The symptom durations and scores showed slight superiority of group B over group A, although these differences were not statistically significant. There were no differences in adverse events. The Shi-cha capsule is efficacious and safe for the treatment of patients with wind-cold type common cold. Larger trials are required to fully assess the benefits and safety of this treatment for common cold.

## 1. Introduction

The common cold is frequent both in the developed and developing countries with the symptom of nasal congestion and discharge, sneezing, mild fever, cough, headache, and malaise, and it is caused by over 200 viruses or bacteria [1–3]. Although the courses are usually benign and self-limited for approximately one week, the high incidence and clinical presentation cost substantial economic burden, due to work absence, physician visit, and over-the-counter drug consumption [4]. It is estimated that an adult may encounter 2 to 4 episodes of common cold every year and physician visit is prompted by clear nasal discharge and congestion, and cough by most patients [5]. Despite the burden of common cold, no effective etiological treatment and little preventive strategy have been proven. Therefore, current therapy focuses on symptom relief. Although various drugs are available in the market to address the symptoms of cold, solid evidence for recommended therapy is rare due to the quality of studies, adverse effects, or disappointing results [6–13].

Traditional Chinese medicine (TCM) is a 3000-year-old holistic system of medicine that combines medical herbs, acupuncture, food therapy, massage, and therapeutic exercise for both extensive treatment and prevention of diseases, including common cold. Dissatisfaction with treatments offered by Western medicine has led many patients to turn to for treating common cold in China and other parts of the world, but the quality of reported studies is of great concern [14]. TCM is a unique system with special etiology and theories for the treatment of common cold according to TCM signs such as avertion to cold, clear nasal discharge, arthralgia of extremities, fever, headache, stuffy nose, sneezing, spiritlessness and weakness, tongue proper, and tongue fur. According to the above symptoms, common cold is categorized as wind-cold type, wind-heat type, as well as summer-heat and dampness type. Among these types, wind-cold type was primarily characterized by avertion to cold and clear nasal discharge, which is treated with “disperse wind-evil and dispel cold as well as supplement *Qi *(vital energy) for strengthening exterior” according to the fundamental principles of TCM.

Shi-Cha capsule (SCC) is a new traditional Chinese prescription and manufactured by the Yunnan Institute of Materia Medica, Yunnan, China. It composes of Shi Jiaocao (*Boenninghausenia sessilicarpa*), Xiao Shancha (*Elsholtzia bodinieri Van*), Huang Qi (*Astragalus membranaceus*), Yu Xingcao (*Houttuynia cordata Thunb*), Qian Liguang (*Climbing groundsel Herb*), as well as Qiang Huo (*Forbes Notopterygium*). Preclinical pharmacologic experiment and secondary stage clinical trial proved that it has the action of disperse wind-evil and dispel cold as well as supplement *Qi* forstrengthening exterior. In addition, the toxicologic study showed that it has no evident adverse-toxic effect. Here we report the findings of a multicenter, prospective, double-blind, randomized, placebo controlled, dose-escalation phase II clinical trial to determine the therapeutic efficacy and safety of the SCC in the treatment of patients with wind-cold type common cold.

## 2. Patients and Methods

### 2.1. Study Design

A multicenter, prospective, randomized, double-blind, placebo-controlled, dose-escalation phase II clinical trial was designed to determine the therapeutic efficacy and safety of 0.6 g and 1.2 g of SCC given three times daily for 3 days to patients with wind-cold type common cold. The protocol was reviewed and approved by the independent ethics committees at West China Hospital of Sichuan University (no. TCM-2010-03). In addition, the trial was registered with the Chinese Clinical Trial Registry (no. ChiCTR-TRC-12002296) and was conducted in accordance with the Good Clinical Practice Guidelines and the Declaration of Helsinki [15]. This trial was also authorized by the SFDA of China (no. 2008L11136). All herbs used in this trial were recognized as safe for use by the State Food and Drug Administration (SFDA) of China. We reported the outcomes according to the Consolidated Standards of Reporting Trials statement [16].

### 2.2. Eligibility Criteria

A total of 377 eligible patients were recruited from five centers in China between November 2010 and March 2011. All patients were examined by one of the clinical study respiratory experts and were enrolled into the study according to the inclusion and exclusion criteria described in Table 1. All patients gave written informed consent prior to participation. A flowchart illustrating the study procedure is provided in Supplementary material 1 (see Supplementary Material available online at doi:10.1155/2012/254571).

### 2.3. Randomization and Blinding

A total of 360 of eligible patients met the inclusive criteria and were randomized into group A (0.6 g × 2 capsules of SCC plus 0.6 g × 2 capsule of placebo), group B (1.2 g × 4 capsules of SCC), or group C (1.2 g × 4 capsules of placebo). 120 patients were required placed into each treatment group. Randomization was conducted in blocks of five in a 1 : 1 : 1 ratio using the PRCO PLAN function of the analysis system of SAS (version 6.12 for Windows) by an independent provider not involved in this study. The randomization lists were placed in two sealed envelopes, and the details were unknown to the study investigators and the patients throughout the course of the study. One envelope was kept by Yunnan Institute of Materia Medica (Yunan Province, China), and the other was kept at the study centers to be opened in case of medical emergency.

### 2.4. Treatment Schedule

All randomized patients in group A were required to take 0.6 g of SCC plus 0.6 g placebo three times daily for 3 days; those in group B took 1.2 g of SCC three times daily for 3 days; and those in group C took 1.2 g of placebo three times daily for 3 days. Patients with common cold were diagnosed with “wind-cold type” (which was an inclusion criteria), the corresponding management was designed to disperse wind-evil and dispel cold as well as supplement *Qi *for strengthening exterior. Built on the fundamental principle of TCM, the SCC preparation used in this study contained six primary herbs (Table 2) that were supplied by Yunnan Institute of Materia Medica. The placebo capsule, the main ingredient was starch, was indistinguishable from the SCC in form, color, taste, size, and packaging. All packages were dispensed by an independent research staff member in a separate room after the visit with the respiratory expert.

### 2.5. Outcome Measures

Patients completed the symptom questionnaire from baseline to day 10 after treatment. These data provided an assessment of all symptom duration, main symptom duration, minor symptom duration, main symptom score, minor symptom score, and cumulative symptom score. The questionnaire consisted of eight symptoms: avertion to cold, clear nasal discharge, arthralgia of extremities, fever, headache, stuffy nose, sneezing, and spiritlessness and weakness. The first two symptoms were main symptom for which the patients provided a graded score (not at all = 0, mild = 3, moderate = 6, severe = 9). The last six symptoms were minor symptom for which the patients provided a graded score (not at all = 0, mild = 1, moderate = 2, severe = 3). The cumulative symptom score was the main symptom score plus the minor symptom score. In addition, tongue proper, tongue fur, and pulse were also assessed (see Supplementary Material 2).

### 2.6. End Points

The primary end point was defined as duration of all symptom. The secondary end points were main symptom duration, minor symptom duration, the changes in main symptom score, minor symptom score, and cumulative symptom score 4 days after the treatment, as well as adverse events. Duration was defined as the number of hours from study enrollment to the last day before the patient answered “No” to the question “Do you think that you are still sick today?”

### 2.7. Followup

In light of the study procedure, patients were seen by a respiratory expert at baseline, day 4, and day 10. During each visit, patients were interviewed by the respiratory expert to ascertain symptoms, compliance, and occurrence of adverse events. In addition, they were in contract with the enrolling research assistant and respiratory expert by telephone throughout the study except for interview. Adherence was assessed by capsule counts and by the daily questionnaire (which asked the patients whether they had taken their capsules and how many capsules they had taken). Adverse events and compliance were monitored. Clinical laboratory evaluation, including routine blood, urine, and stool tests along with hepatic and renal functions, and electrocardiogram, were examined at baseline and at day 4 after treatment to assess the safety of the treatment used for each group.

### 2.8. Sample Size Determination

Based on our experience with TCM in treating common cold and on other similar studies in the literature [17, 18], we assumed the average efficacy *π* = 80%, noninferiority/equivalence boundary value *δ* = −0.15. The sample size of each group was 88 according to the formula *n* = 2[*π*(1 − *π*)*δ* ^−2^](*z*_1−*α*_ + *z*_1−*β*_)^2^ [19]. We allowed for a dropout rate of approximately 10%. Therefore, 360 common cold patients were needed for this study.

### 2.9. Statistical Analysis

All data were documented with Epidata 3.0 by two independent individuals, and the files were locked. The intent-to-treat (ITT) population included all randomized patients who received study medication and attended at least one study visit after the start of treatment. Per-protocol (PP) analysis included all randomized patients who completed study medication and followedup after the start of treatment. The analysis of efficacy was performed in the ITT and PP populations. All quantitative data were expressed as mean ± standard deviation (SD), and one-way analysis of variance (ANOVA) was used to compare the data. A Chi-square test or Fisher's exact test was performed to calculate differences in qualitative data between the three groups. A Kruskal-Wallis *H* Rank-Sum test was performed to calculate differences in rank data between the three groups. The all symptom duration, main symptom duration, and minor symptom duration were estimated by using the Kaplan-Meier technique and were compared by using the stratified log-rank test. A *P* value of <0.05 was considered to indicate statistical significance. Data were analyzed using the statistical software Intercooled Stata version 8.2 for Windows (Stata Corporation, College Station, TX, USA).

## 3. Results

### 3.1. Demographic Data and Baseline Characteristics

**Figure 1:**
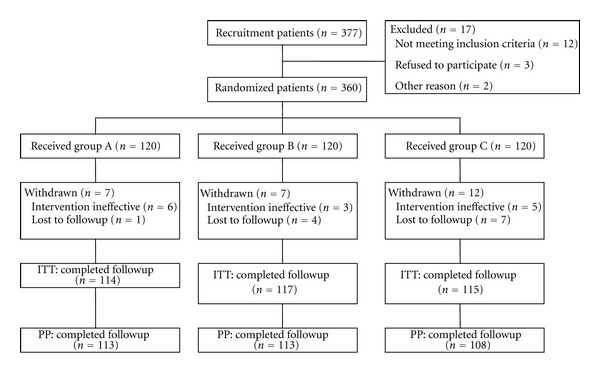
Flow chart of patient disposition. ITT: intent-to-treat; PP: per-protocol analysis.

A total of 377 patients were recruited during a period of five months from November 2010 to March 2011, 360 of these patients met the inclusive criteria, and 120 patients were placed into each treatment group. A total of 26 patients (7.22%) withdrew during the course of the trial: 7 (5.83%) in group A, 7 (5.83%) in group B, and 12 (10%) in group C, respectively (*P* = 0.35). Six patients were withdrawn from the trial because the intervention was ineffective during the treatment period, and 18 patients were withdrawn from the trial because they were lost to followup (Figure 1). There were no significant differences in baseline characteristics in terms of sex ratio, age, weight, height, duration of common cold, body temperature, main symptom score, and cumulative symptom score among the three groups (Table 3).

### 3.2. All Symptom Duration, Main Symptom Duration, and Minor Symptom Duration

**Figure 2:**
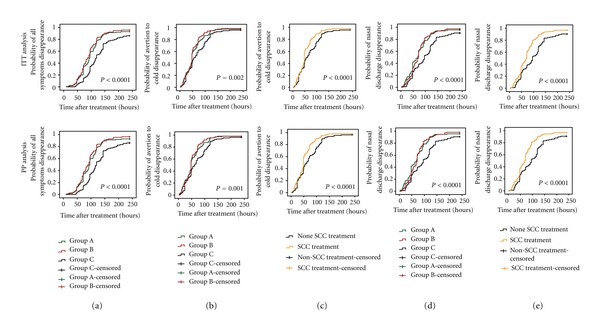
The duration of symptom for patients with wind-cold type common cold receiving 0.6 g SCC (group A), 1.2 g SCC (group B), or 1.2 g placebo (group C). (a) All symptom duration; (b) and (c) the duration of avertion to cold; (d) and (e) the nasal discharge.

For cohort patients, there was a significant difference in all symptom duration and main symptom duration among three Groups both the ITT and PP analyses (Figures 2(a), 2(b), and 2(d); *P* < 0.001). Besides, there was a significant difference in minor symptom duration of arthralgia of extremities, stuffy nose, sneezing, and spiritlessness and weakness among three groups with both the ITT and PP analyses (data not shown, *P* < 0.05). In addition, all symptom duration and main symptom duration showed a slight superiority of group B over group A, although these differences were not statistically significant.

In addition, the main symptom duration and minor symptom duration were determined between patients with SCC treatment and without. There was a significant difference in main symptom duration (Figures 2(c) and 2(e); *P* < 0.0001) and minor symptom duration of arthralgia of extremities, stuffy nose, sneezing, as well as spiritlessness and weakness between patients with SCC treatment and without both the ITT and PP analyses (data not shown, *P* < 0.0001).

### 3.3. Cumulative Symptom Score, Main Symptom Score, and Minor Symptom Score

**Figure 3:**
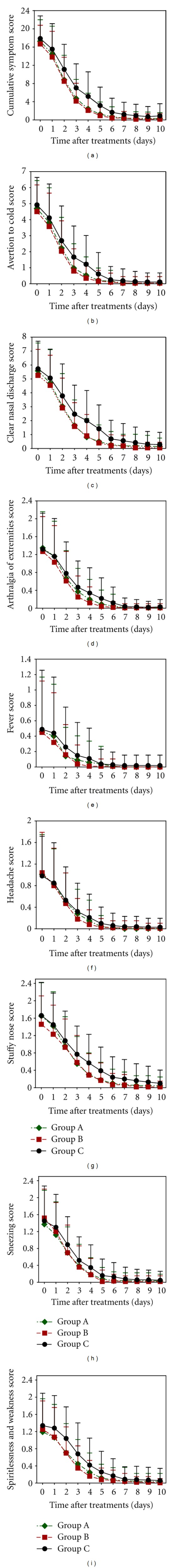
The symptom score for patients with wind-cold type common cold receiving 0.6 g SCC (group A), 1.2 g SCC (group B), or 1.2 g placebo (group C). (a) The cumulative symptom score; (b) the avertion to cold score; (c) the clear nasal discharge score; (d) the arthralgia of extremities score; (e) the fever score; (f) the headache score; (g) the stuffy nose score; (h) the sneezing score; and (i) the spiritlessness and weakness score.

In addition to evaluate the duration of symptom, the symptom score was also compared among three Groups. An improvement in cumulative and individual symptom score was observed from baseline to day 10 in all three groups. The changes from baseline of symptom score at day 4 were compared because the most significant changes in cumulative symptom score and individual symptom score occurred at day 4 after treatment among three groups (Figure 3).

Compared with patients in group C, the ITT and PP analyses showed that patients in groups A and B had significant improvement in change from baseline of cumulative symptom score, main symptom score, and minor symptom score at day 4 (*P* < 0.0001). In addition, the scores showed a slight superiority of group B over group A at day 4, although these differences were not statistically significant (Table 4).

### 3.4. Safety

There were no deaths during the study. There were one, one, and two patients with adverse effects in group A, group B, and group C, respectively (*P* = 0.774). All patients developed light upper abdominal discomfort accompanied by nausea and vomiting. In these patients, we reduced the dose of the experimental drugs and the symptom gradually eased in the absence of any treatment. In addition, no clinically significant changes were noted in laboratory evaluations or physical examinations. We concluded that there was no significant correlation between the use of SCC and adverse events.

## 4. Discussion

To our knowledge, this is the first large randomized, prospective, double-blind, placebo-controlled, dose-escalation trial designated to evaluate the efficacy and safety of TCM in an adult population from China. Our study demonstrated that SCC was efficacious and safe for the management of wind-cold type common cold. Patients receiving 0.6 g and 1.2 g of SCC treatment three times daily demonstrated significant improvement in the symptom duration and change from baseline of symptom score compared to patients receiving the placebo at day 4. Patients receiving 1.2 g of SCC three times daily showed a slight benefit in their symptom duration and change from baseline of symptom score over patients receiving 0.6 g of SCC three times daily during treatment, although this difference was not statistically significant.

To date, there are no validated strategies for the treatment of common cold, and current therapy focuses on symptom relief. This has led to high patient dissatisfaction and frustration with current treatments for common cold. Thus, there is a need for simple, safe, and effective first-line therapies to treat the multiple symptoms of patients with this disorder. TCM stresses differentiation of symptoms and signs; it considers the human being to be an organic entirety and treatment should be emphasized on the entirety. Besides, TCM counts on the synergetic effects of the herbs, which have a general spectra of action. Therefore, TCM practitioners usually use a substantial number of herbs to treat various conditions including common cold, and this will overcome the shortcomings of Western medicine.

TCM theory holds that lung is in charge of skin and hair and keeps the dispersing function, skin and hair damage will result in dysfunction of lung with defensive *Qi*, and lung fails to disperse and descend, eventually leading to common cold. Wind-cold type common cold is mainly manifested as aversion to cold, clear nasal discharge, arthralgia of extremities, fever, headache, stuffy nose, sneezing, spiritlessness and weakness, pale tongue, white fur, and floating pulse according to the fundamental principles of TCM. In this study, wind-cold type common cold patients with were selected for treatment, and the herbal recipe of the SCC, which claimed to disperse wind-evil and dispel cold as well as supply *Qi* forstrengthening exterior, was designed for these patients according to these principles.

The SCC formula contains six herbs including *Boenninghausenia sessilicarpa*, *Elsholtzia bodinieri Van*, *Astragalus membranaceus*, *Houttuynia cordata Thunb*, *Climbing groundsel Herb*, and *Forbes Notopterygium*. After the outbreak of severe acute respiratory syndrome (SARS) in late 2002, TCM has attracted more attention from researchers who endeavor to search for effective antiviral agents and it may be a good candidate with special characteristics for an antivirus [20]. The SCC formula concurred with published pharmacological data which showed that TCM formula can inhibit growth of a variety of virus as well as bacteria [21, 22]. In addition, several previous studies were consistent with our results which showed that TCM formula might be able to improve symptoms more than placebo for patients with common cold [17, 18, 23–25]. However, all studies to date have had important limitation including lack of objective validated outcome measures for common cold [26]. In this study, the duration of symptom and symptom score were used to evaluate the efficacy of SCC, which found not only a significant benefit in shortening the duration symptom, but also decrease of the symptom score from the treatment of SCC for patients with wind-cold type common cold.

Popular opinion confirms that the general public believes TCM to be safe, to cause fewer side effects, and to be less likely to cause dependency. In our study, only two patients who were receiving 0.6 g and 1.2 g of SCC three time daily reported light upper abdominal pain, respectively. Therefore, we concluded that there was no significant correlation between the use of the SCC and adverse events. Nevertheless, many herbs can be toxic, especially in high quantities and with frequent use. Furthermore, herb-synthetic drug interactions can be problematic. Hence, the analysis of adverse effects of TCM for treating common cold is indeed very important.

This study had some potential weaknesses. Only SCC formula for the treatment of common cold with wind-cold type was determined in this study, the outcomes could not be generalized to other herbal formula for the treatment of common cold or other types. The formulation and dosage of SCC standardized, which might not fully reflect the normal practice of TCM which often alters the formula by removing or adding specific herbs according to the patient's body constitution. However, this finding supports the premise that it was of sufficient study to assess the benefit and safety of TCM for the treatment of common cold.

In conclusion, during a treatment period of 3 days, the SCC significantly improved the symptom duration and the changes in symptom score in patients with wind-cold type common cold. Patients receiving 1.2 g of SCC three times daily showed a slight benefit of these end points over patients receiving 0.6 g of SCC three times daily during treatment, although this difference was not statistically significant. Larger trials are required to fully assess the benefits and safety of the use of the two dosages of the SCC for treating wind-cold type common cold.

## Supplementary Material

Supplementary Material 1: The flow chart of study procedures. Summary, all patients were examined by one of the clinical study respiratory experts and were enrolled into the study according to the inclusion and exclusion criteria described. In light of the study procedure, patients were seen by a respiratory expert at baseline, day 4, and day 10. During each visit, patients were interviewed by the respiratory expert to ascertain symptoms, compliance, and occurrence of adverse events. In addition, they were in contract with the enrolling research assistant and respiratory expert by telephone throughout the study except for interview.
Supplementary Material 2: The symptom questionnaire. Summary, Patients completed the symptom questionnaire from baseline to day 10 after treatment. These data provided an assessment of all symptom duration, main symptom duration, minor symptom duration, main symptom score, minor symptom score, and cumulative symptom score. The questionnaire consisted of eight symptoms: avertion to cold, clear nasal discharge, arthralgia of extremities, fever, headache, stuffy nose, sneezing, and spiritlessness and weakness. The first two symptoms were main symptom for which the patients provided a graded score (not at all = 0, mild = 3, moderate = 6, severe = 9). The last six symptoms were minor symptom for which the patients provided a graded score (not at all = 0, mild = 1, moderate = 2, severe = 3). The cumulative symptom score was the main symptom score plus the minor symptom score. In addition, tongue proper, tongue fur, and pulse were also assessed

## Figures and Tables

**Table 1:** Inclusion and exclusion criteria.

Inclusion criteria	
(1) 18 to 65 years of age	
(2) Diagnosis of common cold by a respiratory expert according to relevant criteria and the syndrome criteria of wind-cold type in TCM^†^	
(3) Patient within 48 hours of onset of common cold-like illness	
(4) Patient must be able to understand and give written informed consent and report adverse events and concomitant medication for the duration of the study	

Exclusion criteria	

(1) Patient has suffered from acute viral pharyngitis or laryngitis, acute herpetic pharyngitis or laryngitis, acute conjunctivitis, as well as acute tonsillitis, and so forth	
(2) Patient has taken any medication for relief of symptoms prior to study initiation	
(3) Patient who has fever (>38.5°C)	
(4) Patient who is on analgesic or anti-inflammatory regimen requiring treatment with analgesics, nonsteroidal anti-inflammatory drugs, or steroids	
(5) Patient is pregnant, nursing, or a woman of childbearing potential not practicing adequate contraception. Women, who are uncertain if they are pregnant, may participate in the study, if they undergo a pregnancy test, which shows a negative result	
(6) Patient has comorbid condition, uncontrolled metabolic condition or psychiatric condition that might make tolerance or evaluation of the symptoms difficult	

^†^TCM: traditional Chinese medicine.

**Table 2:** Standard formula of SCC.

Chinese name	Pharmaceutical name	The principle of prescription	Source	Pharmacological actions in TCM
Shi Jiaocao	*Boenninghausenia sessilicarpa *	Primary ingredients in a prescription	The dried whole plant of *Boenninghausenia sessilicarpa Levl *	To dispel wind and dry dampness, to regulate *Qi* to relieve pain, and anti-inflammation caused by common cold
Xiao Shancha	*Elsholtzia bodinieri Van *	Minister herb	The dried whole plant of *Elsholtzia bodinieri Van *	To relieve exterior by diaphoresis, to clear away heat-dampness and promote diuresis, to regulate *Qi* to ease the stomach caused by common cold
Huang Qi	*Astragalus membranaceus *	Minister herb	The dried root of *Astragalus membranaceus *	To tonify *Qi* and relieve exterior, to supply *Qi *for strengthening exterior, to induce diuresis to remove edema, to promote pus discharge and tissue regeneration by strengthening *Qi* caused by common cold
Yu Xingcao	*Houttuynia cordata Thunb *	Adjuvant and messenger herb	The dried whole plant of *Houttuynia cordata Thunb *	To clear away heat-toxin caused by common cold
Qian Liguang	*Climbing groundsel Herb *	Adjuvant and messenger herb	The dried whole plant of* Climbing groundsel Herb *	To clear away heat-toxin caused by common cold
Qiang Huo	*Forbes Notopterygium *	Adjuvant and messenger herb	The dried rhizome and root of *Forbes notopterygium *	To remove dampness to relieve pain caused by common cold

SCC: Shi-Cha capsule; TCM: traditional Chinese medicine; *Qi*: vital energy.

**Table 3:** Demographic data and baseline characteristics: intent-to-treat analysis.

	Group A (*n* = 114)	Group B (*n* = 117)	Group C (*n* = 115)	*χ* ^2^/*F*	*P* values
Sex ratio (male/female)	71/43	76/41	75/40	0.263	0.877^†^
Age (years) (mean ± SD)	35.98 ± 12.453	36.12 ± 12.465	35.64 ± 12.135	0.046	0.955^‡^
Body weight (kg) (mean ± SD)	61.12 ± 10.514	60.23 ± 8.855	58.98 ± 9.827	1.387	0.251^‡^
Height (cm) (mean ± SD)	164.63 ± 8.223	164.36 ± 7.750	164.11 ± 8.266	0.118	0.889^‡^
Body temperature (°C) (mean ± SD)	36.95 ± 0.451	36.90 ± 0.481	36.98 ± 0.507	0.855	0.426^‡^
Course (hours) (mean ± SD)	22.75 ± 11.096	24.21 ± 12.104	23.42 ± 11.477	0.516	0.772^¶^
Main symptoms score (mean ± SD)					
Avertion to cold	4.68 ± 1.741	4.59 ± 1.698	4.85 ± 1.713	1.477	0.478^¶^
Nasal discharge	5.58 ± 1.991	5.26 ± 1.844	5.69 ± 2.002	2.836	0.242^¶^
Minor symptoms score (mean ± SD)					0.478^¶^
Arthralgia of extremities	1.33 ± 0.816	1.26 ± 0.770	1.28 ± 0.779	0.298	0.861^¶^
Fever	0.45 ± 0.705	0.40 ± 0.732	0.49 ± 0.788	1.080	0.583^¶^
Headache	1.01 ± 0.735	1.04 ± 0.781	0.97 ± 0.725	0.492	0.782^¶^
Stuffy nose	1.66 ± 0.762	1.47 ± 0.677	1.64 ± 0.752	4.082	0.130^¶^
Sneezing	1.38 ± 0.803	1.52 ± 0.690	1.42 ± 0.827	2.338	0.311^¶^
Spiritlessness and weakness	1.18 ± 0.771	1.25 ± 0.684	1.29 ± 0.758	1.102	0.576^¶^
Cumulative symptoms score (mean ± SD)	17.27 ± 4.700	16.79 ± 4.333	17.63 ± 4.979	0.962	0.383^‡^

^†^Chi-square test.

^‡^One-way analysis of variance.

^¶^Kruskal-Wallis *H* test.

**Table 4:** Intent-to-treat and per-protocol analyses of main symptom score, minor symptom score, and cumulative symptom score.

Intent-to-treat analysis	Change from baseline to day 4 (mean ± SD)		
	Group A (*n* = 114)	Group B (*n* = 117)	Group C (*n* = 115)
Main symptom score	8.842 ± 3.453	8.513 ± 3.050	7.330 ± 4.209
* P* value versus group C	<0.0001	<0.0001	
Minor symptom score	5.851 ± 2.839	6.120 ± 2.443	5.157 ± 2.648
* P* value versus group C	<0.0001	<0.0001	
Cumulative symptom score	14.320 ± 5.577	13.890 ± 4.949	11.920 ± 6.090
* P* value versus group C	<0.0001	<0.0001	

Per-protocol analysis	Group A (*n* = 113)	Group B (*n* = 113)	Group C (*n* = 108)

Main symptom score	8.920 ± 3.365	8.522 ± 3.094	7.417 ± 4.212
* P* value versus group C	<0.0001	<0.0001	
Minor symptom score	5.876 ± 2.838	6.053 ± 2.394	5.241 ± 2.696
* P* value versus group C	<0.0001	<0.0001	
Cumulative symptom score	14.420 ± 5.498	13.850 ± 5.016	12.060 ± 6.143
* P* value versus group C	<0.0001	<0.0001	

## References

[1] Simasek M, Blandino DA (2007). Treatment of the common cold. *American Family Physician*.

[2] Heikkinen T, Järvinen A (2003). The common cold. *The Lancet*.

[3] Monto AS (1995). Viral respiratory infections in the community: epidemiology, agents, and interventions. *American Journal of Medicine*.

[4] Turner RB (1997). Epidemiology, pathogenesis, and treatment of the common cold. *Annals of Allergy, Asthma and Immunology*.

[5] Woodwell DA, Cherry DK (2004). National Ambulatory Medical Care Survey: 2002 summary. *Advance Data*.

[6] Lissiman E, Bhasale AL, Cohen M (2009). Garlic for the common cold. *Cochrane Database of Systematic Reviews*.

[7] de Sutter AI, van Driel ML, Kumar AA, Lesslar O, Skrt A (2012). Oral antihistamine-decongestant-analgesic combinations for the common cold. *Cochrane Database of Systematic Reviews*.

[8] Albalawi ZH, Othman SS, Alfaleh K (2011). Intranasal ipratropium bromide for the common cold. *Cochrane Database of Systematic Reviews*.

[9] Singh M (2001). Heated, humidified air for the common cold. *Cochrane Database of Systematic Reviews*.

[10] Douglas RM, Hemilä H, Chalker E, Treacy B (2007). Vitamin C for preventing and treating the common cold. *Cochrane Database of Systematic Reviews*.

[11] Wu T, Zhang J, Qiu Y, Xie L, Liu GJ (2007). Chinese medicinal herbs for the common cold. *Cochrane Database of Systematic Reviews*.

[12] Linde K, Barrett B, Wölkart K, Bauer R, Melchart D (2006). Echinacea for preventing and treating the common cold. *Cochrane Database of Systematic Reviews*.

[13] Arroll B, Kenealy T (2005). Antibiotics for the common cold and acute purulent rhinitis. *Cochrane Database of Systematic Reviews*.

[14] Wu T, Yang X, Zeng X, Poole P (2008). Traditional Chinese medicine in the treatment of acute respiratory tract infections. *Respiratory Medicine*.

[15] (2000). World Medical Association Declaration of Helsinki: ethical principles for medical research involving human subjects. *Journal of the American Medical Association*.

[16] Moher D, Schulz KF, Altman D (2005). The CONSORT Statement: revised recommendations for improving the quality of reports of parallel-group randomized trials 2001. *Explore*.

[17] Ji K, Chen J, Li M (2009). Comments on serious anaphylaxis caused by nine Chinese herbal injections used to treat common colds and upper respiratory tract infections. *Regulatory Toxicology and Pharmacology*.

[18] Wang L, Zhang RM, Liu GY (2010). Chinese herbs in treatment of influenza: a randomized, double-blind, placebo-controlled trial. *Respiratory Medicine*.

[19] Hwang IK, Morikawa T (1999). Design issues in noninferiority/equivalence trials. *Drug Information Journal*.

[20] Wang C, Cao B, Liu Q-Q (2011). Oseltamivir compared with the Chinese traditional therapy maxingshigan-yinqiaosan in the treatment of H1N1 influenza: a randomized trial. *Annals of Internal Medicine*.

[21] Li A, Xie Y, Qi F (2009). Anti-virus effect of traditional Chinese medicine Yi-Fu-Qing granule on acute respiratory tract infections. *Bioscience Trends*.

[22] Wang Y, Wang T, Hu J (2011). Anti-biofilm activity of TanReQing, a traditional Chinese Medicine used for the treatment of acute pneumonia. *Journal of Ethnopharmacology*.

[23] Wong W, Lam CLK, Fong DYT (2012). Treatment effectiveness of two Chinese herbal medicine formulae in upper respiratory tract infections-a randomized double-blind placebo-controlled trial. *Family Practice*.

[24] Wang L, Zhang RM, Zhao YL (2008). A multiple center, randomized, controlled, double-blinded and double-dummy trial of Yiqing Shuangjie Capsule and Tablet in treating acute upper respiratory tract infection with the syndrome of heat attacking the lung and Weifen. *Zhong Xi Yi Jie He Xue Bao*.

[25] Chang J, Zhang Y, Mao B, Wang L, Li TQ, Zhang RM (2007). A double-blind, randomized controlled trial of Chaige Qingre Granule in treating acute upper respiratory tract infection of wind heat syndrome. *Zhong Xi Yi Jie He Xue Bao*.

[26] Barrett BP, Brown RL, Locken K, Maberry R, Bobula JA, D’Alessio D (2002). Treatment of the common cold with unrefined Echinacea: a randomized, double-blind, placebo-controlled trial. *Annals of Internal Medicine*.

