# Supplementary material for: Treatment of Common Cold Patients with the Shi-Cha Capsule: A Multicenter, Double-Blind, Randomized, Placebo-Controlled, Dose-Escalation Trial
Source: Evid Based Complement Alternat Med. 2012 Dec 27;2012:254571. doi: 10.1155/2012/254571 (PMC3544370; doi:10.1155/2012/254571)
Supplement: Supplementary file 1 — Supplementary Material 1: The flow chart of study procedures. Summary, all patients were examined by one of the clinical study respiratory experts and were enrolled into the study according to the inclusion and exclusion criteria described. In light of the study procedure, patients were seen by a respiratory expert at baseline, day 4, and day 10. During each visit, patients were interviewed by the respiratory expert to ascertain symptoms, compliance, and occurrence of adverse events. In addition, they were in contract with the enrolling research assistant and respiratory expert by telephone throughout the study except for interview. Supplementary Material 2: The symptom questionnaire. Summary, Patients completed the symptom questionnaire from baseline to day 10 after treatment. These data provided an assessment of all symptom duration, main symptom duration, minor symptom duration, main symptom score, minor symptom score, and cumulative symptom score. The questionnaire consisted of eight symptoms: avertion to cold, clear nasal discharge, arthralgia of extremities, fever, headache, stuffy nose, sneezing, and spiritlessness and weakness. The first two symptoms were main symptom for which the patients provided a graded score (not at all = 0, mild = 3, moderate = 6, severe = 9). The last six symptoms were minor symptom for which the patients provided a graded score (not at all = 0, mild = 1, moderate = 2, severe = 3). The cumulative symptom score was the main symptom score plus the minor symptom score. In addition, tongue proper, tongue fur, and pulse were also assessed [file 254571.f1.pdf]

Supplementary material 1 Flow Chart of Study Procedures

Visit	Times after treatments (days)		
	Baseline	Day 4	Day 10
Acquisition of basic medical history			
Diagnosis of common clod with wind-cold type established by respiratory expert	×		
Inclusion/exclusion criteria confirmed	×		
Written informed consent	×		
Randomization	×		
Fill in general information	×		
Past medical history and treatment history	×		
The combined disease	×		
Medical examination	×	×	×
Combined drug	×	×	×
Efficacy of observation			
Individual symptom score	×	×	×
Main symptom score			
Minor symptom score			
Cumulative symptom score	×	×	×
Safety evaluations			
Blood	×	×	
Urine	×	×	
Stool	×	×	
Liver and renal functions	×	×	×
Electrocardiogram (ECG)	×	×	
Investigation project			
Chest radiography	×		
Pregnancy test	×		
Reporting the adverse events		×	×
Others			
Supply or recovery of investigational product to the patients	×	×	×
Medication accountability assessed		×	×

Supplementary material 2 Symptom Questionaire

Hospital No:		Date:	Patient No:	Patient name:	Diagnosis:	Patient address:
Clinical Symptom		Score	Not at all [†]	Mild	Moderate	Severe
Main symptom	Avertion to cold		None	Slight, no need of extra clothes	Need of extra clothes	Need of thick clothes or blanket
	Nasal discharge		None	Occasional nasal discharge	Nasal discharge	Large amount of nasal discharge
Main symptom score						
			Not at all [‡]	Mild	Moderate	Severe
Minor symptom	Arthralgia of extremities		None	Slight arthralgia of extremit	Arthralgia of extrem	Incapable of flexing and extendi
	Fever		None	37.3-37.5 ℃	37.6-38℃	≥ 38.1˚
	Headache		None	Slight and occasional	Lasting	Severe and unable to
	Stuffy nose		None	Slight, no breathing difficu	Congestion and not smooth breathing	Obvious with mouth assistan
	Sneezing		None	Occasional	Between slight and severe	Frequent
	Spiritlessness and weakne		None	Malaise	Difficult but able to work	Unable to work
Cumulative symptom score						
Tongue and pulse						
Tongue and pulse	Tongue proper		Pale tongue proper □ others □			
	Tongue fur		White fur on tongue □ others □			
	Pulse		Floating pulse □ others □			

[†] Not at all = 0 score; Mild = 3 scores; Moderate = 6 scores; Severe = 9 scores.

[‡] Not at all = 0 score; Mild = 1 score; Moderate = 2 scores; Severe = 3 scores.
